# 2543. Multisite Analysis of Vancomycin Predictions in a Bayesian Software in Patients with and without Acute Kidney Injury in Adult and Pediatric Patients

**DOI:** 10.1093/ofid/ofad500.2160

**Published:** 2023-11-27

**Authors:** Maria-Stephanie Hughes, Jasmine Hughes

**Affiliations:** InsightRX, Boston, MA; InsightRX, Boston, MA

## Abstract

**Background:**

Inpatients that require vancomycin therapy often present with an acute kidney injury (AKI). Due to their unstable renal function, pharmacists hesitate to base dosing decisions on predictions provided by pharmacokinetic (PK) models within a Bayesian software. This study will therefore compare accuracy of predictions by various PK models in patients presenting with and without AKI.

**Methods:**

Adult and pediatric patients receiving vancomycin therapy from 1/1/2022 - 12/31/22 that were dosed using a Bayesian software were retrospectively evaluated. Data on patient age, weight, height, serum creatinine (SCr), sex, and vancomycin doses and levels were recorded. Baseline SCr was defined as the median of SCrs before vancomycin was given. AKIs at start of therapy were defined as a baseline SCr or any SCr collected < 12 hours into therapy that was > 1.5 times higher than any subsequent SCrs. *A priori* accuracy was calculated by measuring root mean square error (RMSE) of 7 PK models for patients with and without AKIs at start of therapy. Statistical difference between RMSE values was determined by non-overlapping 95% confidence intervals.

**Results:**

A total of 149,100 adult and 4,311 pediatric patient treatment courses were included (see Table 1). Higher RMSE, or lower accuracy, was seen in the AKI groups for all models evaluated, and all except one were statistically different (see Table 2). The RMSE in the group without an AKI at baseline ranged from 4.27 - 6.70 mg/L whereas in the AKI group it ranged from 4.58 - 7.31 mg/L. The absolute difference between groups for each model ranged from 0.03 - 2.05 mg/L and the percent difference ranged from 0.45-39%.

**Figure:**
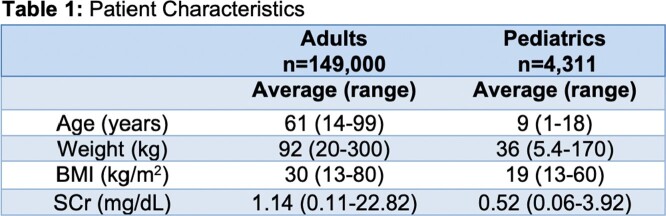

**Figure:**
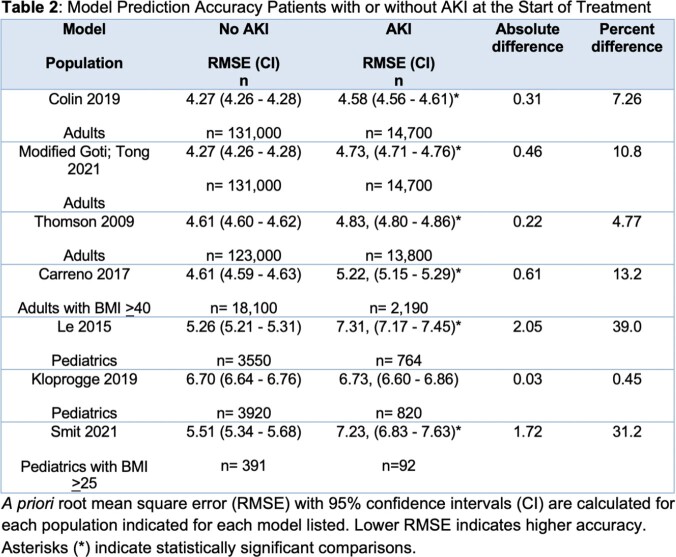

**Conclusion:**

PK models had reduced accuracy in patients with an AKI at start of therapy compared to those that do not. However, differences were small and statistical differences were likely detected due to large sample sizes. For adults, the Colin and Thomson models showed small (< 10%) reductions in accuracy in patients with an AKI, while for pediatric patients, the Kloprogge model showed equal accuracy in all patients regardless of AKI status. These findings may help pharmacists decide whether to use PK models to inform initial vancomycin dosing.

**Disclosures:**

**Maria-Stephanie Hughes, PharmD**, InsightRX: Stocks/Bonds **Jasmine Hughes, PhD**, InsightRX: Employee

